# Screening of TNFα, IL-10 and TLR4 single nucleotide polymorphisms in individuals with asymptomatic and chronic cutaneous leishmaniasis in Colombia: a pilot study

**DOI:** 10.1186/s12879-017-2281-4

**Published:** 2017-02-28

**Authors:** Angélica Mera-Ramírez, Andrés Castillo, Yenifer Orobio, María Adelaida Gómez, Carolina Gallego-Marin

**Affiliations:** 1 Centro Internacional de Entrenamiento e Investigaciones Médicas-CIDEIM, Carrera 125 #, 19-225 Cali, Colombia; 2 Departamento de Biología. Facultad de Ciencias Naturales y Exactas, Universidad del Valle, Calle 13 No, 100-00 Cali, Colombia; 3 Division of Infectious Diseases and Immunology, University of Massachusetts Medical School, 55 Lake Avenue North, 01655 Worcester, MA USA

**Keywords:** Cutaneous leishmaniasis, Single nucleotide polymorphism, Asymptomatic infection, Chronicity

## Abstract

**Background:**

Clinical manifestations of cutaneous leishmaniasis (CL) caused by *Leishmania (Viannia)* range from asymptomatic infection to self-limited, or chronic (non-healing) cutaneous lesions. Given the critical role of the immune response in the clinical outcome of CL, it is plausible that functional polymorphisms in immune-related genes contribute to define the clinical manifestations of human infection.

**Methods:**

DNA samples from a retrospective cohort of individuals from an endemic area of *L. V. panamensis* transmission in Colombia were used to determine the frequency of SNPs in *TNFα*, *IL-10* and *TLR4* genes. DNA samples were obtained from 74 adult participants: 38 patients presenting chronic cutaneous leishmaniasis (CCL) and 36 individuals with asymptomatic infection. Genotyping of TNFα-308G/A, IL-10-819C/T, and TLR4 Asp299Gly and Thr399Ile SNPs, was conducted by PCR-restriction fragment length polymorphisms. Allele, genotype frequencies and associations between SNPs and clinical groups were evaluated.

**Results:**

The A allele in TNFα-308G/A SNP was found more frequently in individuals with asymptomatic infection (16% vs 7%), whereas the CC genotype in IL-10-819 C/T SNP was more frequent in patients with CCL (34% vs. 27% in asymptomatic individuals). No differences in allele frequencies for TLR4 SNPs were found among groups.

**Conclusion:**

This study provides a reference base for statistical power calculation and design of association studies of genetic polymorphisms in immune response related-genes and the pathogenesis of infections caused by *L. V. panamensis.*

## Background

Cutaneous leishmaniasis (CL) caused by *Leishmania* of the *Viannia* subgenus is characterized by a wide spectrum of clinical manifestations. Infection can result in self-limited lesions that resolve without treatment, or chronic (non-healing) lesions lasting several years and often not responding to conventional chemotherapy [1, 2]. In addition, asymptomatic infection occurs in a variable but often high proportion of residents of endemic areas for CL [2, 3].

Polarization of Th1/Th2 responses has been shown to determine resistance and susceptibility in murine models of *Leishmania major* infection [4, 5]. However, the factors and mechanisms that drive the different outcomes of human infections are not fully understood. Although the immunological profile associated with disease susceptibility or resistance to *L. Viannia* infection remains unknown, correlations between pro- and anti-inflammatory cytokine production and clinical manifestations have been observed. High TNFα production has been shown to contribute to parasite control in early stages of *L. V. braziliensis* infection, and exacerbation in later stages [1, 6, 7]. Higher levels of TNFα and IL-10 secretion are detected after in vitro recall responses of mononuclear cells from individuals with chronic and recurrent *L. V. panamensis* infection, compared to asymptomatic individuals (Navas, A. et.al, unpublished observations). Likewise, high levels of IL-10 have been detected in individuals with active and chronic CL caused by *L.V. braziliensis* [8–10], while low secretion has been described during asymptomatic infection or spontaneous healing [11].

Toll-like receptors (TLR)-ligand interactions initiate signal transduction cascades that subsequently result in transcription of inflammatory mediators and cytokines such IL-10 and TNFα [12]. TLR4 has been involved in the control of *Leishmania* growth in experimental murine leishmaniasis [13–16]. We have previously reported the involvement of TLR4 in TNFα production in human macrophages in response to *L.V. panamensis* infection, and its participation in the early control of infection [17]. Given the critical role of immune mediators in the pathogenesis and clinical outcome of CL caused by *L. Viannia*, it is plausible that functional polymorphisms in immune-related genes contribute to define the outcome of human infection.

Susceptibility to mucosal disease caused by *L.V. braziliensis* has been associated with a single nucleotide polymorphism (SNP) in the promoter region of the *TNFα* gene (-308G/A), mediating higher levels of cytokine production [18, 19]. Similarly, high levels of IL-10 have been associated with cutaneous lesion development during *L.V. braziliensis* infection, and have been linked to the presence of the -819C/T SNP in the IL-10 promoter [10]. The polymorphisms 896A/G and 1196C/T (commonly named Asp299Gly and Thr399Ile, respectively) in the coding region of the *TLR4* gene affect the extracellular domain of the receptor impairing the ligand-binding or protein interactions [20]. These variants have been described as a risk factor for septic shock, infection with Gram-negative bacteria and development of severe malaria [20–22]. Genotyping of Asp299Gly and Thr399Ile SNPs in CL caused by *L. major* showed a higher frequency (40.9%) in patients with chronic disease compared to individuals with asymptomatic infection (13.3%) suggesting the involvement of TLR4 in susceptibility and severity of CL [23].

The aim of this pilot study was to determine the frequency of SNPs in *TNFα*, *IL-10* and *TLR4* genes in individuals with clinical and immunological evidence of *Leishmania* infection in endemic areas of *L. V. panamensis* transmission in Colombia, and to explore its relationship with the clinical outcome of infection.

## Methods

### Study design, subjects and sampling

From a database of stored human biological samples with a total of 560 records, collected from years 2007 to 2010, a retrospective pilot case-control study was designed to explore the frequencies of SNPs in immune related genes and the clinical outcome of *L. Viannia* infection, and included CCL patients (cases) and individuals with asymptomatic infection (controls). Inclusion criteria for individuals with asymptomatic infection were defined as a positive in vitro lympho-proliferation assay in response to *Leishmania* antigen [24] and no evidence or history of dermal lesions suggestive of CL. Patients with CCL were included if they presented cutaneous lesions with ≥ 4 months of disease evolution, parasitological confirmation of infection by microscopic examination of lesion smears or biopsy specimens and/or parasite isolation, and/or positive Montenegro skin test reaction (MSR) or lympho-proliferation assay, who had not received anti-leishmanial treatment before enrollment. All subjects were of Afro-Colombian ethnicity.

This study used DNA samples obtained from buccal swabs (BuccalAmp, Epicentre) from patients with CCL (*n* = 38) and from individuals with asymptomatic infection (*n* = 36), all residents of endemic areas of *L.V. panamensis* transmission in rural areas of Tumaco, Colombia. DNA samples were obtained from the CIDEIM Biobank. Demographics of study participants are shown in Table 1.

**Table 1:** Baseline characteristics of the study groups

Characteristic	Asymptomatic infection (*n* = 36)	Chronic cutaneous leishmaniasis (*n* = 38)
Age-years. Mean (range)	43 (17–67)	31 (9–69)
Sex:		
Male; % (*n*)	58.3 (21)	55.3 (21)
Female; % (*n*)	41.7 (15)	44.7 (17)
Positive lympho-proliferation assay; % (*n*)	100 (36)	15.8 (6)
Montenegro skin test zone, mm. Mean (range)	N/A	13.25 (10–18)
Number of lesion; median (range)	N/A	2.2 (1–10)
Time of evolution of older lesion, months; median (range)	N/A	16.3 (4–72)
Type of lesions: % (*n*)		
Ulcer	N/A	71.1 (27)
Plaque	N/A	5.3 (2)
Nodule	N/A	7.9 (3)
Not determined	N/A	15.8 (6)
Patients from whom *Leishmania* strains were isolated % (*n*)	N/A	34.2 (13)
*L. V. panamensis* % (*n*)	N/A	100 (13)
Not determined^a^	N/A	65.8 (25)

*Abbreviations*: *N/A* not applicable

^a^Not determined because no strain was isolated

### DNA genotyping

One hundred nanograms of genomic DNA were used for PCR amplification. Genotyping of TNFα-308G/A (Reference SNP-rs1800629), IL-10-819C/T (rs1800871), and TLR4, Asp299Gly (A/G) (rs4986790) and Thr399Ile (C/T) (rs4986791) was conducted by PCR-restriction fragment length polymorphisms (RFLP) as described previously [10, 23, 25, 26]. Primer sequences, restriction endonucleases and fragment sizes are presented in Table 2. Digestion of PCR products was analyzed by electrophoresis on a 3% agarose gel stained with ethidium bromide. Quality control of restriction enzymes was conducted using PCR amplification products of the *β-globin* gene from DNA isolated from THP-1 or U937 cells.

**Table 2:** Primer sequences and restriction endonucleases used for RFLP analysis and fragment sizes for identification of SNPs

*SNP*	*Primers (5’-3’)*	*Restriction endonuclease*	*Fragment sizes (bp)*
*IL-10*-819 C/T	F, 5- TCA TTC TAT GTG CTG GAG ATG G -3	*Mae* III	CC: 125 + 84 TT: 209 TC: 209 + 125 + 84
	R, 5- TTT GGG GGA AGT GGG TAA GAG T -3		
*TNF-α-*308 G/A	F, 5- AGG CAA TAG GTT TTG AGG GCC AT -3	*Nco*I	GG: 96 + 20 AA: 116 GA: 116 + 96 + 20
	R, 5- ACA CTC CCC ATC CTC CCT GCT -3		
*TLR4* A/G (Asp299Gly)	F, 5- GAT TAG CAT ATC TAG ACT ACT ACC TCC ATG -3	*I Nco*I	AA: 248 GG: 222 + 26 GA: 248 + 222 + 26
	R, 5- GAT CAA CTT CTG AAA AAG CAT TCC CAC -3		
*TLR4* C/T (Thr399Ile)	F, 5 -GGT TGC TGT TCT CAA AGT GAT TTT GGG AGA A- 3	*Hinf*I	CC: 406 TT: 380 + 26 CT: 406 + 380 + 26
	R, 5 -ACC TGA AGA CTG GAG AGT GAG TTA AAT GTT- 3		

### Statistical analysis

Allele and genotype frequencies were calculated for both study groups (CCL patients and asymptomatic individuals) by direct counting. Fisher’s exact test or Chi-square test was used to determine Hardy-Weinberg equilibrium (HWE) and the differences between allele and genotype frequencies, depending on the calculated expected frequencies. Odds ratios and 95% confidence intervals were calculated using logistic regression models to assess the magnitude of association between SNPs and clinical groups. Analyses were done using the STATA SE 12.1 software, except for differences in minor allele frequencies (MAF), which were calculated using the prop.test function on R 3.1.1 software (URL http://www.R-project.org/). *p* values < 0.05 were considered statistically significant.

## Results

DNA samples from a total of 74 participants were analyzed. Amplification products for TNFα genotyping were obtained from 83.7% of samples (62 of 74), for IL-10 from 72.9% (54 of 74), and for TLR4 Asp299Gly and Thr399Ile from 89.1% of samples (66 of 74). Samples from which PCR amplification products were not obtained were excluded from further analysis, and thus, the denominator for calculation of genotype and allele frequencies for each SNP was variable. The distribution of the genotypic variants for TNFα-308G/A and TLR4 Asp299Gly SNPs met the HWE (Fisher’s exact test, *p* values: 0.650 and 1 respectively), whereas IL-10-819C/T did not, (*X* ^2^ test, *p <* 0.05). Determination HWE for the TLR4 Thr399Ile SNP was not possible due to the absence of the T allele within the study population. Distribution of allele and genotype frequencies is summarized in Table 3.

**Table 3:** Genotype, allele frequency and MAF of *TNFα*, *IL-10* and *TLR4* gene polymorphisms in individuals with chronic and asymptomatic cutaneous leishmaniasis

SNPs		Frequency; *N* (%)		*P* value	OR (95% CI)
		Asymptomatic	CCL		
TNFα-308					
Genotype	AA	1 (4)	0 (0)	0.45 ^b^	0 (0–0)
	GG	20 (71)	29 (85)	0.06 ^a^	0.39 (0.14–1.05)
	GA	7 (25)	5 (15)	0.30 ^a^	1.93 (0.54–0.94)
Allele	A	9 (16)	5 (7)	0.13 ^a^	2.41 (0.76–7.67)
	G	47 (87)	63 (93)	0.13 ^a^	0.41 (0.13–1.32)
MAF		0.161	0.074	0.126 ^a^	
IL-10-819					
Genotype	CC	6 (27)	11 (34)	0.21 ^a^	0.49 (0.16–1.51)
	TT	9 (41)	16 (50)	0.51 ^a^	0.69 (0.23–2.07)
	CT	7 (32)	5 (16)	0.16 ^a^	2.52 (0.68–9.33)
Allele	C	19 (43)	27 (42)	0.92 ^a^	1.04 (0.48–2.26)
	T	25 (57)	37 (58)	0.92 ^a^	0.96 (0.44–2.09)
MAF		0.568	0.578	0.918 ^a^	
TLR4 Asp299Gly					
Genotype	AA	28 (85)	28 (85)	0.45 ^b^	1.25 (0.38–4.22)
	GG	0 (0)	0 (0)	–	–
	AG	5 (15)	5 (15)	0.63 ^b^	1.00 (0.20–4.88)
Allele	A	61 (92)	61 (92)	0.63 ^b^	1.00 (0.22–4.58)
	G	5 (8)	5 (8)	0.63 ^b^	1.00 (0.22–4.58)
MAF		0.076	0.076	1.000 ^a^	
TLR4 Thr399Ile					
Genotype	CC	34 (100)	34 (100)	–	–
	TT	0 (0)	0 (0)	–	–
	CT	0 (0)	0 (0)	–	–
Allele	C	68 (100)	68 (100)	–	–
	T	–	–	–	–
MAF		0.000	0.000	–	

^a^ Chi square test

^b^ Fisher exact test (one-tail)

The frequencies of the G allele in the −308 position of the promoter region of the *TNFα* gene was similar between the study groups, while the A allele was found more frequently in individuals with asymptomatic infection (16% vs. 7%) (Table 3). This was concordant with a higher frequency of the heterozygous genotype found in asymptomatic individuals (25%) compared to patients with chronic CL (15%). Genotyping of the IL-10-819 C/T SNP showed that the CC genotype was more frequent in patients with CCL (34% vs. 27% in asymptomatics), while higher frequency of the heterozygous genotype was found in asymptomatic individuals (32% vs. 16%). Despite this, no significant differences in allele, genotype frequencies or MAF values in TNFα-308G/A or IL-10-819C/T SNPs were found between the study groups (Table 3).

Analysis of TLR4 Asp299Gly SNP showed identical frequencies between groups for the AA and AG genotypes (85 and 15%, respectively). The GG genotype was not found in any of the study participants. All individuals presented the homozygous CC genotype at position 1196 (Thr399Ileu) of the *TLR4* gene. Since the 1196 CT and TT genotypes were not found in any participant, comparison between groups, MAF and co-segregation analysis with TLR4 Asp299Gly SNP could not be performed.

The presence of more than one SNP per individual was observed in 6 asymptomatic individuals and in 7 CCL patients. No relationship between the presence of multiple SNPs in one individual and the clinical outcome of infection could be established.

## Discussion

Polymorphisms in promoter and coding regions of cytokine genes have shown associations with the clinical outcome and immunological responses during *Leishmania* infections [10, 27]. The TNFα-308A allele has been associated with increased gene expression and thereby protein production, exacerbated inflammatory responses and tissue damage [28]. An association between the TNFα-308G/A polymorphism and risk of development of mucosal disease has been shown during *L. V. braziliensis* infections in Venezuelan CL patients [1, 6, 19]. In contrast, no relationship could be established between this SNP and asymptomatic, self-healing or CCL caused by *L. major* in an Iranian population [27]. The divergence in the frequency of the TNFα-308A allele between studies could reflect distinct mechanisms of pathogenicity and host responses resulting from infections with different *Leishmania* species [19, 27]. The observed frequencies of the A allele in CCL patients (7%) and asymptomatic individuals (16%) are comparable to those reported for the same clinical groups (7.8 and 11.4%, respectively) in a larger *Leishmania* infected population in Iran (*N* = 150) [27]. Despite the differences in allele frequency, no association with the clinical outcome of CL could be determined.

Functional studies have demonstrated that the -819C/T SNP in the IL-10 promoter plays a role in up-regulation of IL-10 and modification of NF-κB binding. The CC genotype has been associated with higher levels of IL-10 production [10]. Presence of the C allele has been related to increased risk of lesion development (OR = 2.5 (1.12–5.7), *p* < 0.003) in *L.V. braziliensis* infection [10]. High levels of IL-10 production have been described in active and chronic CL [8–10], while low cytokine levels have been related to self-healing and asymptomatic infection caused by *L.V. braziliensis* [11] and *L.V. panamensis* (Navas, A., unpublished observations). Although our results did not establish significant differences in the frequency of IL-10-819C/T SNP between the study groups, a higher frequency of the -819CC genotype in CCL patients was observed, potentially suggesting a contribution to pathogenicity.

Genetic variants in TLR genes correlate with disease severity of multiple infectious diseases [29]. For TLR4, two frequently co-segregating polymorphisms (Asp299Gly and Thr399Ile) have been shown to increase the risk of sepsis [30], Gram-negative infections [31] and severe malaria [22]. The two polymorphic sites have been predicted to affect ligand and co-receptor binding regions, respectively [20]. Higher frequency of the Asp299Gly polymorphism has been reported in patients with CCL caused by *L. major* compared to patients with acute disease, associating this SNP to increased risk of disease severity [23]. In contrast, no relationships between these functional TLR4 polymorphisms and susceptibility to visceral leishmaniasis in an Iranian population could be established [26]. The homozygous GG genotype for the Asp299Gly SNP was not found among our study groups comprised of an Afro-descendant population of the South-pacific region of Colombia, although a frequency of 0.7 to 1.5% has been reported in a Colombian population in the North-Center region of the country [32]. The absence of the TT genotype for the Thr399Ile SNP in our study group is concordant with absence of this genotype in other Colombian populations.

## Conclusions

Leishmaniasis is a complex disease and multiple host genes are involved in the outcome of infection. Polymorphisms in four genes were analyzed in an attempt to overcome the limitations of single gene polymorphism studies reported previously in CL. However, only 13 individuals showed more than one SNP limiting the power of a multi-marker SNP analysis. Results presented here provide a reference base for design of prospective, power-defined genetic studies to explore the relationship between immune-related genes (either individually or as multi-marker signatures), disease outcome and pathogenesis of infections caused by *L. V. panamensis.*

## Abbreviations

CCL: Chronic Cutaneous Leishmaniasis
CL: Cutaneous Leishmaniasis
HWE: Hardy-Weinberg Equilibrium
MAF: Minor Allele Frequency
MSR: Montenegro skin test reaction
RFLP: Restriction Fragment Length Polymorphisms
SNP: Single Nucleotide Polymorphism
TLR: Toll-Like Receptor

## References

[1] Ribeiro-De-Jesus A, Almeida RP, Lessa H, Bacellar O, Carvalho EM (1998). Cytokine profile and pathology in human leishmaniasis. Braz J Med Biol Res.

[2] Weigle KA, Santrich C, Martinez F, Valderrama L, Saravia NG (1993). Epidemiology of cutaneous leishmaniasis in Colombia: environmental and behavioral risk factors for infection, clinical manifestations, and pathogenicity. J Infect Dis.

[3] Carvalho EM, Correia Filho D, Bacellar O, Almeida RP, Lessa H, Rocha H (1995). Characterization of the immune response in subjects with self-healing cutaneous leishmaniasis. Am J Trop Med Hyg.

[4] Sacks D, Noben-Trauth N (2002). The immunology of susceptibility and resistance to Leishmania major in mice. Nat Rev Immunol.

[5] Scott P, Natovitz P, Coffman RL, Pearce E, Sher A (1988). CD4+ T cell subsets in experimental cutaneous leishmaniasis. Mem Inst Oswaldo Cruz.

[6] Bacellar O, Lessa H, Schriefer A, Machado P, Ribeiro de Jesus A, Dutra WO, Gollob KJ, Carvalho EM (2002). Up-regulation of Th1-type responses in mucosal leishmaniasis patients. Infect Immun.

[7] Da-Cruz AM, de Oliveira MP, De Luca PM, Mendonca SC, Coutinho SG (1996). Tumor necrosis factor-alpha in human american tegumentary leishmaniasis. Mem Inst Oswaldo Cruz.

[8] Castellano LR, Filho DC, Argiro L, Dessein H, Prata A, Dessein A, Rodrigues V (2009). Th1/Th2 immune responses are associated with active cutaneous leishmaniasis and clinical cure is associated with strong interferon-gamma production. Hum Immunol.

[9] Maurer-Cecchini A, Decuypere S, Chappuis F, Alexandrenne C, De Doncker S, Boelaert M, Dujardin JC, Loutan L, Dayer JM, Tulliano G (2009). Immunological determinants of clinical outcome in Peruvian patients with tegumentary leishmaniasis treated with pentavalent antimonials. Infect Immun.

[10] Salhi A, Rodrigues V, Santoro F, Dessein H, Romano A, Castellano LR, Sertorio M, Rafati S, Chevillard C, Prata A (2008). Immunological and genetic evidence for a crucial role of IL-10 in cutaneous lesions in humans infected with Leishmania braziliensis. J Immunol.

[11] Gomes-Silva A, de Cassia Bittar R, Dos Santos Nogueira R, Amato VS, da Silva Mattos M, Oliveira-Neto MP, Coutinho SG, Da-Cruz AM (2007). Can interferon-gamma and interleukin-10 balance be associated with severity of human Leishmania (Viannia) braziliensis infection?. Clin Exp Immunol.

[12] Akira S, Takeda K (2004). Toll-like receptor signalling. Nat Rev Immunol.

[13] Kropf P, Freudenberg MA, Modolell M, Price HP, Herath S, Antoniazi S, Galanos C, Smith DF, Muller I (2004). Toll-like receptor 4 contributes to efficient control of infection with the protozoan parasite Leishmania major. Infect Immun.

[14] Kropf P, Freudenberg N, Kalis C, Modolell M, Herath S, Galanos C, Freudenberg M, Muller I (2004). Infection of C57BL/10ScCr and C57BL/10ScNCr mice with Leishmania major reveals a role for Toll-like receptor 4 in the control of parasite replication. J Leukoc Biol.

[15] Ribeiro-Gomes FL, Moniz-De-Souza MC, Alexandre-Moreira MS, Dias WB, Lopes MF, Nunes MP, Lungarella G, Dosreis GA (2007). Neutrophils activate macrophages for intracellular killing of Leishmania major through recruitment of TLR4 by neutrophil elastase. J Immunol.

[16] Whitaker SM, Colmenares M, Pestana KG, Mcmahon-Pratt D (2008). Leishmania pifanoi proteoglycolipid complex P8 induces macrophage cytokine production through Toll-like receptor 4. Infect Immun.

[17] Gallego C, Golenbock D, Gomez MA, Saravia NG (2011). Toll-Like Receptors Participate in Macrophage Activation and Intracellular Control of Leishmania (Viannia) panamensis. Infect Immun.

[18] Blackwell JM (1999). Tumour necrosis factor alpha and mucocutaneous leishmaniasis. Parasitol Today.

[19] Cabrera M, Shaw MA, Sharples C, Williams H, Castes M, Convit J, Blackwell JM (1995). Polymorphism in tumor necrosis factor genes associated with mucocutaneous leishmaniasis. J Exp Med.

[20] Arbour NC, Lorenz E, Schutte BC, Zabner J, Kline JN, Jones M, Frees K, Watt JL, Schwartz DA (2000). TLR4 mutations are associated with endotoxin hyporesponsiveness in humans. Nat Genet.

[21] Lorenz E, Mira JP, Cornish KL, Arbour NC, Schwartz DA (2000). A novel polymorphism in the toll-like receptor 2 gene and its potential association with staphylococcal infection. Infect Immun.

[22] Mockenhaupt FP, Cramer JP, Hamann L, Stegemann MS, Eckert J, Oh NR, Otchwemah RN, Dietz E, Ehrhardt S, Schroder NW (2006). Toll-like receptor (TLR) polymorphisms in African children: common TLR-4 variants predispose to severe malaria. Proc Natl Acad Sci U S A.

[23] Ajdary S, Ghamilouie MM, Alimohammadian MH, Riazi-Rad F, Pakzad SR (2011). Toll-like receptor 4 polymorphisms predispose to cutaneous leishmaniasis. Microbes Infect.

[24] Bosque F, Saravia NG, Valderrama L, Milon G (2000). Distinct innate and acquired immune responses to Leishmania in putative susceptible and resistant human populations endemically exposed to L. (Viannia) panamensis infection. Scand J Immunol.

[25] Paskulin DD, Fallavena PR, Paludo FJ, Borges TJ, Picanco JB, Dias FS, Alho CS (2011). TNF -308G > a promoter polymorphism (rs1800629) and outcome from critical illness. Braz J Infect Dis.

[26] Rasouli M, Keshavarz M, Kalani M, Moravej A, Kiany S, Badiee P (2012). Toll-like receptor 4 (TLR4) polymorphisms in Iranian patients with visceral leishmaniasis. Mol Biol Rep.

[27] Kamali-Sarvestani E, Rasouli M, Mortazavi H, Gharesi-Fard B (2006). Cytokine gene polymorphisms and susceptibility to cutaneous leishmaniasis in Iranian patients. Cytokine.

[28] Wilson AG, Symons JA, McDowell TL, Mcdevitt HO, Duff GW (1997). Effects of a polymorphism in the human tumor necrosis factor alpha promoter on transcriptional activation. Proc Natl Acad Sci U S A.

[29] Schroder NW, Schumann RR (2005). Single nucleotide polymorphisms of Toll-like receptors and susceptibility to infectious disease. Lancet Infect Dis.

[30] Lorenz E, Mira JP, Frees KL, Schwartz DA (2002). Relevance of mutations in the TLR4 receptor in patients with gram-negative septic shock. Arch Intern Med.

[31] Agnese DM, Calvano JE, Hahm SJ, Coyle SM, Corbett SA, Calvano SE, Lowry SF (2002). Human toll-like receptor 4 mutations but not CD14 polymorphisms are associated with an increased risk of gram-negative infections. J Infect Dis.

[32] Zafra G, Florez O, Morillo CA, Echeverria LE, Martin J, Gonzalez CI (2008). Polymorphisms of toll-like receptor 2 and 4 genes in Chagas disease. Mem Inst Oswaldo Cruz.

